# Neuropsychological Function and Quality of Life after Resection of Suspected Lower-Grade Glioma in the Face Primary Motor Area

**DOI:** 10.3390/jcm10040580

**Published:** 2021-02-04

**Authors:** Mattias Stålnacke, Tommy Bergenheim, Rickard L. Sjöberg

**Affiliations:** Department of Clinical Science—Neurosciences, Umeå University, 901 85 Umeå, Sweden; Tommy.bergenheim@umu.se (T.B.); rickard.sjoberg@umu.se (R.L.S.)

**Keywords:** glioma resections, quality of life, onco-functional balance, anxiety, awake craniotomy

## Abstract

The negative side effects of neurosurgical resection of the lower third of the primary motor cortex (M1) are often described as relatively mild. However, detailed descriptions of how these resections affect neurocognitive function, speech, mental health and quality of life (QoL) are sparse. In the present study, seven patients with suspected lower-grade glioma (WHO II-III) in the inferior M1 were assessed for facial motor function, cognitive function, anxiety and QoL before and after awake surgical resections. The main finding was that after surgery, six of the seven patients experienced a mild facial motor dysfunction, mainly affecting the mouth, tongue and throat. At the group level, we were also able to observe a significant postoperative decline in maximum verbal speed, whereas no negative effects on measures of word production (i.e., verbal fluency) were seen. Self-reported QoL data suggest that some patients experienced increased social isolation postoperatively but do not lend support to the interpretation that this was caused by direct neurological side effects of the surgery. The results appear to support the general notion that awake surgery in the lower M1 can be performed safely and with postoperative deficits that are most often perceived by the patient as tolerable.

## 1. Introduction

Maximal surgical resection does not typically cure WHO grade II–IV gliomas but appears to prolong patient survival [1,2,3]. On the other hand, extensive surgery, particularly in sensitive areas, may cause neurological deficits. Some of these, particularly surgically induced motor and language deficits, seem to have a negative impact on survival, at least in high-grade tumors [4]. Furthermore, and equally important, such surgically induced deficits may also negatively impact patient quality of life (QoL). Due to this, the importance of optimizing the onco-functional balance (i.e., weighing the benefit of radical resection against the risk of impairing QoL and brain function) is receiving increasing attention [3,5].

Historically, the inferior primary motor cortex (M1), which includes the motor function for the face, tongue and larynx, has been defined by neurosurgeons as “eloquent” [6], and concerns are frequently raised that resections in this area may cause both motor and language deficits. As evidenced from research in patients with central or peripheral facial paresis, motor deficits in the face area may have several profound negative consequences [7]. Such consequences may include negative effects on speech, non-verbal communication and the ability to handle food in the mouth [8]. In addition, between 30% and 40% of patients with severe facial motor dysfunction (i.e., peripheral facial palsy) experience anxiety, some 17% experience depression at clinically significant levels [9] and impaired QoL is common [10]. The relation between psychological distress and the severity of facial motor dysfunction is not linear [11]. That is, in some patients, objectively mild facial motor dysfunction can be associated with significant decreased mental health and QoL.

Currently, studies presenting detailed, standardized evaluations of outcomes for speech, cognitive and motor functions, as well as patient-reported outcomes regarding QoL and mental health, for resection in the lower M1 are missing. However, existing data do suggest that the negative effects of glioma surgery in the lower part of M1 may be mild or negligible for many patients [12].

The most important evidence in this regard comes from Magill et al. [12], who recently presented a retrospective case series of M1 glioma resections that included 28 cases of resections exclusively performed in the lower M1. In these cases, the authors only described 11 (39%) of their patients as having persistent deficits at long-term follow-up with only 3 (10%) of these classified by the authors as “moderate-severe”. Data in this study were, however, collected retrospectively from postoperative clinical notes made by either the neurosurgeon or the neuro-oncologist. No details about the nature of the deficits were given and as noted by the authors, the extent to which these notes may have missed deficits is not known.

Clinical experiences from resections in the lower M1 have also been reported in epilepsy surgery patients. In 1954, Penfield and Jasper [13] proposed that resections of this area could be performed with only minor and transient effects on speech articulation and movement of the lower parts of the face. They furthermore noted that motor function in the face, mouth, tongue and throat appeared to be bilaterally represented for most patients.

Since then, approximately 50 epilepsy surgical resections of the lower M1 have been reported [14,15,16,17,18]. Some measures of cognitive function have briefly been presented in about 30 of these cases [14,19]. The incidence of immediate postoperative dysphasia seems to be approximately 40% and for long-term dysphasia 8% [18]. Other forms of postoperative cognitive dysfunction (mild memory deficits, perceptual problem, slow speech and impaired attention span) have been noted, in single cases [14]. None of these studies reported data on possible changes in mental health or QoL.

In sum, the published and unpublished neurosurgical clinical experiences of lower M1 resections suggest that such procedures can be performed safely with only minor sequelae for the patient. As techniques for the mapping of individual brain function, such as awake craniotomy and functional resonance imaging, become more widely used, surgery in this area is likely to become an increasingly realistic therapeutic option at many neurosurgical centra [20]. However, since even very subtle residual symptoms in face, tongue and speech function may adversely impact QoL, the lack of more systematic, detailed high-resolution studies of such effects is a problematic gap in the clinical literature.

Here, we present an attempt to fill this gap by presenting a prospective study of seven consecutive cases of resections of suspected lower-grade tumors in the inferior part of the primary motor cortex performed between May 2018 and June 2020. The aim of the study was to describe both the patient-reported outcome and the outcome studied by clinical and neuropsychological instruments with particular attention to patients’ facial motor function, speech motor function, language ability, mental health and QoL.

## 2. Materials and Methods

### 2.1. Patient Characteristics

Seven consecutive patients operated by the senior author (RLS) between May 2018 and June 2020 for suspected lower-grade tumors in the inferior primary motor cortex were included in this study. One patient was male and six were females. Four of the tumors were in the right hemisphere and three were in the left hemisphere. Six patients were right-handed and one was left-handed. All surgeries were performed on radiological suspicion, based on preoperative MRI, of lower-grade glioma (WHO II-III). However, in two cases, the diagnosis turned out to be glioblastoma (WHO IV). Pathological anatomical diagnoses for the remaining patients were astrocytoma WHO II (one patient); anaplastic astrocytoma (two patients); and WHO II 1p/19q-positive oligodendroglioma (2 patients). In all tumors except one of the anaplastic astrocytomas, immunohistochemistry revealed an IDH1 R132H mutation. Mean age at time of surgery was 51.2 years (SD = 11.7, range = 38.8–71.3).

### 2.2. Lateralization of Tumor and Verbal Laterality

To establish hemisphere dominance, several different methods were utilized. A laterality index was calculated from an fMRI investigation performed preoperatively. Briefly, this index compares the BOLD signal in the inferior and middle frontal gyrus during speech between hemispheres. The index ranges from −1 to +1: +1 = maximally left-lateralized; −1 = maximally right-lateralized [21]. Positive verbal responses induced by perioperative direct stimulation, handedness and results from a dichotic listening task [22] were also used.

### 2.3. Neuropsychological Function

The cognitive domains of primary interest for this study were verbal and executive function. In addition to this, long-term memory and working memory were also assessed. Verbal function was assessed with the verbal fluency task and executive function with the color word interference task, both presented in the Delis–Kaplan Executive Functions System (D-KEFS) [23]. The Brief Visual Memory Test—Revised (BVMT-R) [24] was chosen as the long-term memory test and the Digit Span test from WAIS-IV [25] was chosen as the test for working memory and attention. The neuropsychological assessment was administered before and after surgery (postoperative, mean = 5.9 month, SD = 7.5, range = 2.2–12.9).

#### 2.3.1. Verbal Fluency

In the letter fluency condition, the patients were asked to generate as many words as possible with a certain initial letter for 60 s. In the semantic fluency condition, the patients were instructed to generate as many words as possible within a verbal category for 60s. The conditions were repeated three times for letter fluency and two times for semantic fluency.

#### 2.3.2. Color Word Interference

All four conditions described by Kaplan et al. [23] were administrated. Presented with sheets of paper containing 50 items, each patient was asked to perform different cognitive tasks as fast as possible but without mistakes. Condition 1: name the color in painted squares. Condition 2: read color names. Condition 3: name the color of the print and not read the name of color words printed in incongruent colors (e.g., the word green printed in blue color). Condition 4: switching between reading color names and naming the color of the print in incongruently colored words.

#### 2.3.3. BVMT-R

A paper with six geometric figures was presented to the subject for 3 × 10 s. After each of the presentations, the subject was asked to draw as many of the designs as possible. After 25 min, the subjects were asked to recall as many designs as possible.

#### 2.3.4. Digit Span

Digits (0–9) were read by the administrator in a random order. The subject was then asked to repeat the digits. The task started with two consecutive digits and added one digit every other item until the subject could no longer repeat the digits in a reliable way. In the second condition, the subjects were asked to repeat the digits in reverse order. The score is an aggregate of the two conditions.

### 2.4. Mental Health, Every Day Executive Function and Mental Fatigue

Questionnaires for mental health, mental fatigue and every day executive function were administrated simultaneously with the neuropsychological assessment before and after surgery.

#### 2.4.1. The Hospital Anxiety and Depression Scales (HADS)

HADS is a screening form for identifying clinically relevant anxiety and depression in non-psychiatric patients. The 14 statements (7 items for anxiety and depression, respectively) are scored on a scale of 0–3 [26].

#### 2.4.2. Behavior Rating Inventory of Executive Function—Adult Version (BRIEF-A)

BRIEF-A includes nine scales representing different parts of executive function in everyday life (Inhibit, Self-Monitor, Plan/Organize, Shift, Initiate, Task Monitor, Emotional Control, Working Memory and Organization of Materials). From these subscales, two indexes are derived: Behavioral Regulation Index (Inhibit, Shift, Emotional Control and Self-Monitor) and Metacognition Index (Working Memory, Plan/Organizing, Task Monitor and Organization of Material). The overall Global Executive Composite can then be calculated adding the Behavioral Regulation and Metacognition Indexes together [27].

#### 2.4.3. Mental Fatigue Scale (MFS)

The 15-item questionnaire encompasses several dimensions that are often affected in patients experiencing mental fatigue, such as sleep and sensory, emotional and cognitive domains, as well as mental recovery and diurnal variation [28].

### 2.5. Quality of Life

#### EORTC QLQ-C30 and BN-20

These questionnaires were used to evaluate effects on overall health-related and symptom-specific QoL [29,30]. QLQ C-30 is a multidimensional questionnaire especially developed with oncology patients in mind, containing subscales for everyday function (Physical, Role, Emotional, Cognitive and Social function), specific physical symptoms (Fatigue, Pain, Nausea and Vomiting) and global health status. BN-20 is an additional module, specific for brain tumor patients, covering four different domains (Future uncertainty, Visual disorder, Communication deficit, Motor dysfunction). The results from the different subscales and domains are transformed to a score between 1 and 100. Patients answered these questionnaires before surgery and approximately 3 months (mean = 3.3 month, SD = 0.5, range = 2.7–3.9) after surgery.

### 2.6. Facial Motor Function

Post-surgery, the patients’ facial motor function was assessed with the House–Brackmann motor function grading system [31] and QoL related to facial motor dysfunction was assessed with the Swedish version of the Facial Clinimetric Evaluation questionnaire (FaCE) [32]. The administration of the questionnaire and the rating of facial motor function were conducted retrospectively at least 2.5 months after surgery (FaCE, mean = 1.2 years, SD = 0.9, range = 0.2–2.3, House–Brackmann, mean = 1.2 years, SD = 0.9, range = 0.2–2.3).

#### 2.6.1. House–Brackmann Facial Nerve Grading Scale

Patients were rated on a six-grade scale (1 = 100% recovery, 6 = 0% recovery) of motor dysfunction and asymmetry in rest and motion for the forehead, eyes and mouth.

#### 2.6.2. Facial Clinimetric Evaluation (FaCE)

The questionnaire addresses, in addition to a global facial function score, six dimensions of facial functions important for QoL (Facial Movement, Facial Comfort, Oral Function, Eye Comfort, Lacrimal Control, Social Function). The scores are transformed for total facial dysfunction and domain ranging from 0 to 100 (0 = worst, 100 = best) and compared to a facial palsy group [32].

### 2.7. Statistical Method

All statistical analyses were conducted with the Statistical Package for the Social Sciences (IBM SPSS Statistics for Windows, Version 25.0, Armonk, NY, USA). For parameters where pre- and postoperative results were available, a paired-sample *t*-test was used, whereas the analyses of postoperative results in comparison with a normative sample of a facial palsy patient used a one-sample *t*-test.

### 2.8. Informed Consent

All surgeries were performed on clinical neuro-oncological indication and all patients gave their informed consent according to the standard clinical procedure. This information included detailed information on the risks and possibilities of surgery in the facial area of the primary motor cortex. Patients were also routinely informed perioperatively when this part of the tumor resection was started, and their consent was renewed at this time.

In addition, all patients gave verbal and written informed consent to participate in this research (collection of pre- and postoperative data for research purposes), including the use of clinical data for research purposes. This research was conducted in accordance with the Declaration of Helsinki and is covered by the following decisions from the regional ethics committee at Umeå, Sweden: Dnr: 2016/479-3, Dnr, 2018-402-32M and Dnr, 2016/200-31.2.10.

### 2.9. Surgical Procedure

All patients were operated in an asleep-awake-awake procedure. With the patient asleep, the dura was opened, and the location of the central sulcus was confirmed by identification of a phase reversal on a medianus sensory evoked potential (SEP) stimulation using a 1 × 4 grid. After this, a 1 × 4 grid was placed along the primary motor cortex and used for monitoring motor evoked potentials in the hand, arm leg and foot. The hand motor area was subsequently identified using motor evoked potentials. After this, the procedure was continued with the patient awake. Speech mapping and deep motor stimulation were performed before, during and at the end of tumor resection. Patient behavior, speech, hand function, swallowing and facial and tongue motor function were continuously monitored by a neuropsychologist (MS) during the procedures. Care was taken to avoid extending surgery into what had been defined as the hand motor area.

In all patients except one, the inferior border of the resection cavity was defined by the sylvian fissure which was exposed by subpial dissection (for JD-75, the distance between the inferior resection cavity and the sylvian fissure was approximately 8 mm). Resection cavities were medially extended to or slightly beyond the insula in all cases except JD-75 and AW-49, for whom the medial border was approximately 6 and 10 mm lateral of the insula, respectively. For all patients, resection cavities were extended to or beyond the precentral and postcentral sulcus, respectively. Mean distance between the lower border of the resection cavity and the superior border (as measured on T1 images approximately 6 months postop for patients with lower-grade glioma and on immediate postop images for patients with glioblastoma) was 26 mm (SD = 5, range = 22–35). In all cases, resections were subtotal to the extent that there were remaining pathological flair signals on immediate postoperative images typically diffusely extending into the insula and/or the hand motor area. However, in three of the five patients with lower-grade glioma (WHO II-III), these signals decreased during the first 6 months postoperatively. In two of the cases (TN-74 and JD-75) with left-sided lesions, stimulation of the face motor area produced significant, transient dysphasic symptoms that resulted in a perioperative decision to leave parts of the tumor for which resection had been planned.

## 3. Results

### 3.1. Patient Dropout

Due to administrative errors, two of the patients (LBS81 and AW67) did not complete their questionnaires for mental health, adaptive everyday function and mental fatigue prior to surgery. One patient (JD75) was excluded from the statistical analysis of QoL since the results from the preoperative administration were suspected to be negatively affected by unrelated health issues.

In some cases, due to time constraints, a few of the tasks in the comprehensive neuropsychological evaluation were not performed. LBS81 has no preoperative results for CWIT1 and AW49 has no preoperative results from CWIT2 and CWIT4 and no postoperative results for BVMT-R and Digit Span.

### 3.2. Facial Motor Function

**Figure 1:**
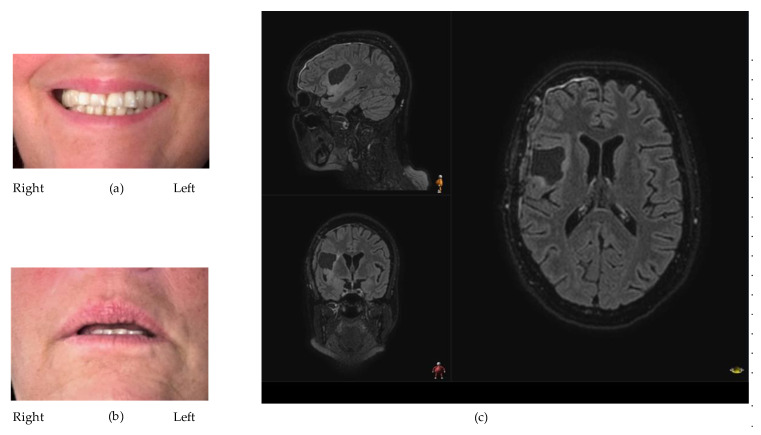
Both the photographs and the MR image (T2 Flair) depict the patient with the most severe facial motor deficit. (**a**) The patient has been instructed to show as big a smile as possible. A slightly less pronounced left furrow can be noticed. During the execution of the smile, the left side was slightly less responsive. (**b**) The patient was instructed to relax the face with a slight separation of the lips. A slight lowering of the left corner of the mouth can be noticed. (**c**) MR image (T2 Flair) of the resection cavity approximately 5 months postop.

Postoperatively, one patient was classified as normal regarding facial motor function according to the House–Brackmann scale (TN74). The remaining patients were classified as having mild dysfunction with slight asymmetries of the mouth (i.e., House–Brackmann score = 2). However, as demonstrated in Figure 1, the effects on the symmetry of the mouth in this group were very subtle.

**Figure 2:**
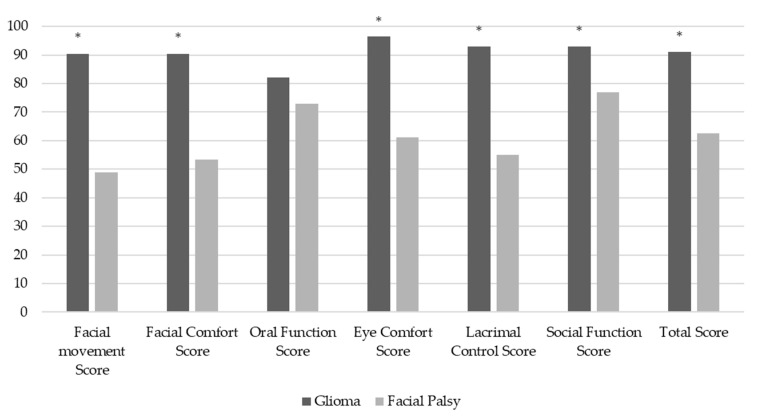
Postoperatively, the glioma group had significantly better QoL scores regarding dimensions affected by facial motor dysfunction in all domains, except oral function, as compared to patients with facial palsy [32]. * = *p* ≤ 0.05.

In the FaCE questionnaire, the glioma group had a mean above 90 in all dimensions except oral function, where the score was 82.1 (Table 1). One patient (SA71) reported that both sides of the face were affected, and two patients reported that one side was affected (JD75, AW49). When compared with published normative data on patients with facial palsy, the patients in this study scored significantly better in all domains except oral function (Figure 2).

### 3.3. Neuropsychological Function

As can be seen in Table 2, there were no significant differences in verbal fluency at the group level. Mean letter fluency was the same pre- and postoperatively at 12 words per minute (wpm) (preoperative mean = 12.2 wpm, SD = 4, range = 6.3–17.3, postoperative M = 12.1 wpm, SD = 3.4, range = 7–17.3). Category fluency was also stable at a slightly higher speed than letter fluency, which is expected (preoperative mean = 21.5 wpm, SD = 5.9, range = 13.5–30.5, postoperative mean = 20.9 wpm, SD = 6.1, range = 14–26).

However, the baseline condition of the color word interference test showed a significant effect on psychomotor speed for reading and pronunciation. That is, the time it took patients to read the names of colors aloud, as well as the time it took to name colors, increased between pre- and postoperative testing. For color naming, the decrease in speed went from 82.9 wpm (SD = 16.8, range = 63.8–100) preoperatively to 71.9 wpm (SD = 12, range = 56.6–88.2) postoperatively. For reading aloud, the decline in speed went from 109.1 wpm (SD = 22.2, range = 83.3–136.4) to 95.5 wpm (SD = 25.8, range = 68.2–142.9).

A similar effect was also found for the switching condition which taxes executive function but also is dependent on psychomotor speed assessed with the baseline conditions. The inhibition condition was not affected.

No significant changes were found in either long-term memory (BVMT-R) or working memory (Digit Span).

### 3.4. Mental Health, Every Day Executive Function and Mental Fatigue

None of the measured domains of mental health (anxiety, depression, mental fatigue) significantly changed (Table 2), but there was a negative, non-significant, trend for self-reported anxiety that went from below the cut-off for clinically relevant anxiety to above the cut-off postoperatively (cut-off = 7, preoperative mean = 5, SD = 4.7, range = 1–13, postoperative mean = 7.4, SD = 4.2, range = 0–12).

#### 3.4.1. Everyday Executive Function

Overall self-rated everyday executive function did not change significantly in any life domain assessed by the BRIEF-A. The Behavioral Regulation Index (BRI) includes the ability to inhibit inappropriate reactions, be flexible to changes and be able to maintain emotional control (preoperative mean = 37.3, SD = 7.9, range = 30–50, postoperative mean = 41, SD = 5.2, range = 32–48, *t* = −1.9, df = 4, *p* = 0.139) and the Metacognitive Index (MI) includes the ability to initiate, plan and organize activities, as well as one’s ability to monitor the effectiveness of one’s own problem solving (preoperative m = 50.8, SD = 9.7, range = 43–67, postoperative mean = 57.3, SD = 10.7, range = 44–74, *t* = −1.7, df = 4, *p* = 0.16). Global executive function (GEF), an aggregate of BRI and MI, did not show any significant change either (preoperative mean = 88, SD = 17.3, range = 76–117, postoperative mean = 98.3, SD = 15, range = 76–117, *t* = −1.9, df = 4, *p* = 0.134).

#### 3.4.2. Quality of Life

With one exception, no statistically significant changes, or meaningful trends, in QoL (EORTC QLQ C30 and BN 20) were found (Table 2). The exception was the dimension social function, where a significant worsening was reported. However, this change was driven by patients LBS 81 and AW 49 who both entered a long-term self-quarantine to protect themselves from COVID-19 between answering the preoperative vs. postoperative questionnaires.

### 3.5. Lateralization of Tumor and Verbal Laterality

When results from fMRI, direct perioperative stimulation, the dichotic listening task and handedness were combined, six of seven patients were classified as left-dominant and one patient was classified as having a bilateral speech representation. In four of the cases, all variables pointed towards a left-sided speech dominance. In two patients (MN58 and AW49), the results from the fMRI lateralization index took precedence over the dichotic listening task which pointed towards a bilateral and left-sided speech lateralization, respectively. In one patient (TN74), direct perioperative stimulation took precedence over ambiguous fMRI results, resulting in an assessment of left-sided speech lateralization. Three of the surgeries were performed in the dominant or bilaterally dominant hemisphere (TN74, JD75, MN58) and four were performed in the non-dominant hemisphere (SA71, AW67, LBS81, AW49).

## 4. Discussion

In 1954, Penfield and Jasper [13] favorably compared their experiences of lower M1 surgery to those of central or peripheral facial palsy. In doing so, they stated that “complete removal of the face area, which we have carried out often, result only in a comparative impairment of movement of the lower face but no interference with movement of forehead and closure of eyes”, and that “Movement of tongue and swallowing are not permanently disturbed by removal of the cortical representation on one side.” Resections of centers for vocalization, the authors noted, did not cause paralysis if performed on one side. Following these authors, several neurosurgeons have continued to perform resections of epileptic foci and gliomas in this area. At the same time, however, this area is often described as “eloquent” in the authoritative neurosurgical literature and surgery in the area is sometimes avoided due to concerns that even slight effects on speech and facial symmetry may negatively influence patient mental health measures and QoL. As evidence demonstrating positive effects of aggressive surgery on survival in high- and low-grade glioma accumulates, the need to thoroughly evaluate and understand effects of resections in this area has thus become increasingly important.

The results of the present prospective study provide some detailed nuance to this discussion that is in line with the general assumption that maximal resections can be performed in this area in centers with appropriate protocols in place. However, even in carefully monitored patients, such resections are not completely devoid of side effects. The most important postoperative finding in the present cohort was a statistically significant decline in fast verbal production (82.9 wpm and faster). The facial movements of the lower face were also, typically, slightly affected. However, effects on speech were minor and speech production in the verbal fluency tasks (requiring a lower rate of verbal production at 21.5 wpm) was not affected by surgery.

With regard to QoL, the EORTC QLQ-C30 and BN20 questionnaires did not reveal any significant differences before vs. after surgery except for the dimension social function which deteriorated considerably in two patients. However, those two answered the follow-up questionnaire while in self-quarantine due to the COVID 19 epidemic. The fact that five of our patients had top scores in the social function dimension of the FaCE scale for QoL (Table 2), where questions are asked specifically about how facial function affects social function, suggests that this change in QoL cannot be explained as a direct sequela of the surgery. Furthermore, no significant differences in self-reported measures of mental health outcomes (executive function, depression/anxiety and mental fatigue) were observed. We did, however, observe a trend towards increased levels of anxiety in the postoperative assessment, in that four of seven patients received scores on the anxiety scale that were above the cut-off for mild but clinically relevant anxiety. The reasons for this increased level of anxiety are not clear, but it suggests that mental health is an important domain to take into consideration after surgery. One aspect that could have had an effect is the over-representation of women in our sample (six women, one male). Women with facial palsy seem to have an increased risk for mental health deterioration [33].

Our patients’ experiences of motor function in the face and mouth area compare favorably to those previously reported for patients with facial palsy (Table 1). This suggests that postoperative effects of a resection of the lower M1 are indeed less troublesome than the experience of facial palsy. However, it should be noted in this context that facial nerve palsy and lower M1 resections do not cause the exact same neurological effects. For instance, due to functional anatomical reasons, the effects on eye function and lacrimal control are more pronounced in facial palsy, whereas resections of lower M1 can be expected to have more negative effects on oral function and swallowing (which is only, to a very limited extent, influenced by the facial nerve). Another reason to treat the comparison between our sample and previously collected data on facial palsy patients with care is of course that they were collected under different circumstances and for different purposes.

Finally, with regard to findings of a lack of changes in QoL, mental health and neuropsychological measures between pre- and postoperative measurements, it is important to note that our sample was underpowered to identify minor or medium-sized changes as statistically significant. That is, whereas positive findings (such as the effects on speech speed and the favorable comparison of these patients to a reference group with peripheral facial palsy) are more likely to reflect “true effects”, any lack of statistically significant findings between pre- and postoperative measurements in other domains, in this small cohort, should be interpreted with caution.

In sum, the general pattern of results presented in the present study seems to confirm the notion that the adverse effects of surgery in this area are mild and well tolerated. One interesting observation that can be made in this context is furthermore that the histopathological diagnosis of the resection material in several patients revealed tumor foci with a higher grade than was initially suspected based on the preoperative MRI. This illustrates another advantage of aggressive surgery in this location.

However, our study also demonstrates that when high-resolution methods such as advanced psychological tests are applied, some residual symptoms can typically be discerned. The most important result of this kind in our study is the negative effect on maximal speech speed. This symptom, which does not appear to be associated with declines in verbal fluency (i.e., the ability to produce and pronounce words belonging to certain categories or starting with certain letters), has not previously been tested (and consequently not described) in this group of patients. However, our results suggest that it may be reasonable to inform patients about the risk of this side effect prior to neurosurgical procedures in the lower M1.

It should furthermore be noted that in two patients with left-sided tumors, the resection was adjusted to avoid effects on speech. This strongly suggests that these surgeries should be performed with awake speech mapping, at least when performed in the dominant hemisphere.

## 5. Conclusions

In sum, the results of pre- and postoperative data in the present cohort of brain tumor patients that had awake surgery in the lower M1, support the general notion, first expressed by Penfield and Jasper, that surgery can be performed safely and with postoperative deficits that are most often perceived by the patient as tolerable. However, our results also suggest that limitation in maximal speech speed is a typical side effect of surgery in this area, even in patients that have otherwise tolerated the procedure well.

## Figures and Tables

**Table 1:** FaCE (Facial Clinimetric Evaluation) and House–Brackmann Facial Nerve Grading Scale (H-B).

	SA71	TN74	JD75	AW67	LBS81	MN58	AW49	Mean	SD	*t*	F-P Mean ^†^	*p*
**FaCE**												
Facial Movement	100	100	91.7	100	91.7	91.7	58.3	90.5	14.8	7.5	48.9	0.000
Facial Comfort	83.3	100	83.3	100	100	100	66.7	90.5	13.1	7.5	53.3	0.000
Oral Function	100	100	62.5	100	75	87.5	50	82.1	20.2	1.2	73	0.277
Eye Comfort	75	100	100	100	100	100	100	96.4	9.5	9.9	61.2	0.000
Lacrimal Control	75	100	100	100	75	100	100	92.9	12.2	8.2	55.1	0.000
Social Function	100	100	75	100	100	100	75	92.9	12.2	3.4	77	0.014
Total	91.7	100	83.3	100	93.3	96.7	71.7	91	10.3	7.3	62.5	0.000
**H-B**	2	1	2	2	2	2	2					

Results from the FaCE questionnaire for quality of life (QoL) related to facial motor function. ^†^ The glioma patients are compared to patients diagnosed with facial palsy in the validation study for the Swedish version of FaCE [32].

**Table 2:** Patient characteristics, QoL, cognitive function and mental health.

Patient ID	SA71		TN74		JD75		AW67		LBS81		MN58		AW49							
	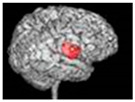		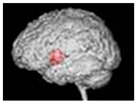		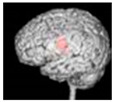		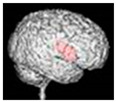		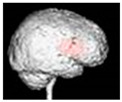		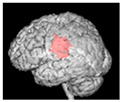		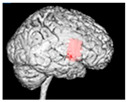							
Hemisphere	Right		Left		Left		Right		Right		Left		Right							
PAD	Anaplastic astrocytoma WHO III		Oligodendroglioma WHO II		Anaplastic oligodendroglioma WHO III		Astrocytoma WHO II-III		Oligodendroglioma WHO II		Glioblastoma WHO IV		Glioblastoma WHO IV		Mean	SD				
Age (y)	47.2		43.7		43.3		51.7		38.8		62.3		71.3		51.2	11.7				
	**SA71 Pre**	**SA71 Post**	**TN74 Pre**	**TN74 Post**	**JD75 Pre**	**JD75 Post**	**AW67 Pre**	**AW67 Post**	**LBS81 Pre**	**LBS81 Post**	**MN58 Pre**	**MN58 Post**	**AW49 Pre**	**AW49 Post**	**Mean Pre**	**SD Pre**	**Mean Post**	**SD Post**	***t***	***p***
**QLQ C30 + BN20**																				
Physical func. ^†^	100		100	100			93.3	92	73.3	100	60	53.3	100	46.7	87.8	17.1	78.4	26.2	0.5	0.621
Role func. ^†^	100	16.7	100	100			83.3	83.3	16.7	0	50	16.7	66.7	33.3	69.5	32.3	41.7	40.5	2.2	0.080
Emotional func. ^†^	33.3	25	41.7	66.7			91.7	66.7	66.7	58.3	75	83.3	83.3	25	65.3	23.2	54.2	24	1	0.387
Cognitive func. ^†^	100	83.3	100	100			100	133.3	83.3	83.3	50	83.3	83.3	16.7	86.1	19.5	77.8	31	0.6	0.562
Social func. ^†^	33.3	16.7	100	83.3			100	100	50	0	83.3	33.3	100	0	77.8	29.2	38.9	43	2.7	0.046
Fatigue ^‡^	33.3	33.3	11.1	0			0	22.2	22.2	33.3	33.3	66.7	11.1	77.8	18.5	13.5	38.9	28.8	−1.8	0.13
Pain ^‡^	50	50	0	0			0	0	66.7	0	16.7	33.3	0	0	22.2	29.2	13.9	22.2	0.7	0.518
Nausea ^‡^	0	16.7	0	0			33.3	0	50	0	0	33.3	0	0	13.9	22.2	8.3	13.9	0.4	0.679
Global health ^†^	58.3	25	8.3	75			33.3	91.7	33.3	66.7	58.3	50	33.3	50	37.5	18.8	59.7	23.2	−1.4	0.218
Future uncertainty ^‡^	83.3	75	33.3	25			8.3	0	41.7	16.7	41.7	66.7	8.3	75	36.1	27.7	43.1	33.1	−0.5	0.633
Visual disorder ^‡^	0	0	0	0			0	0	0	0	11.1	22.2	0	33.3	1.9	4.5	9.3	14.8	−1.4	0.235
Communic. deficit ^‡^	0	22.2	33.3	11.1			0	11.1	11.1	0	44.4	66.7	22.2	33.3	18.5	18.1	24.1	23.8	−0.8	0.49
Motor dysfunc. ^‡^	0	0	0	0			0	0	0	0	33.3	55.6	22.2	44.4	9.3	14.8	16.7	26.1	−1.6	0.175
**Verbal fluency**																				
Letter flu.	30	37	33	27	19	21	30	38	48	52	52	43	45	36	36.7	11.9	36.3	10.1	0.2	0.883
Category flu.	48	49	48	42	27	29	29	33	44	52	61	59	44	28	43	11.8	41.7	12.1	0.4	0.68
**CWIT**																				
Color naming	31	37	47	53	45	51	30	39		34	34	42	30	36	36.2	7.8	41.7	7.5	−12.6	0.000
Reading	22	29	34	44	36	40	24	29	23	21	26	32		25	27.5	5.9	31.4	8.1	−3.1	0.028
Inhibition	55	70	81	93	100	93	60	53	54	55	48	51	59	80	65.3	18.5	70.7	18.4	−1.3	0.235
Switching	56	69	81	99	82	124	60	76	54	60	59	67		122	65.3	12.7	88.1	26.8	−3.2	0.023
**BVMT-R**																				
Total Recall	31	26	28	36	14	19	16	26	27	22	15	24	29		22.9	7.5	25.5	5.8	−1.3	0.251
Delayed Recall	12	12	10	12	6	6	6	10	11	10	8	11	11		9.1	2.5	10.2	2.2	−1.7	0.158
Digit Span	9.5	11	7.5	7	7.5	6.5	6.5	6.5	8	9.5	6	6	7		7.4	1.1	7.8	2	−0.6	0.58
**Mental Health**																				
HAD—Dep.	5	8	0	0	9	6		1		4	1	1	1	4	3.2	3.8	3.4	2.9	−0.5	0.621
HAD—Anx.	5	9	3	7	13	11		0		12	3	4	1	9	5	4.7	7.4	4.2	−1.8	0.147
MFS	2.5	12.5	3.5	3	13	14		5.5		9	2.5	8.5	1.5	14	4.6	4.8	9.5	4.3	−2.3	0.081

^†^ Higher score = higher function, score range: 0–100, ^‡^ higher score = higher dysfunction, score range: 0–100.

## Data Availability

The data presented in this study is available on reasonable request from the corresponding author.
